# Sheathless Shape-Based Separation of *Candida Albicans* Using a Viscoelastic Non-Newtonian Fluid

**DOI:** 10.3390/mi10120817

**Published:** 2019-11-26

**Authors:** Jeonghun Nam, Hyunseul Jee, Woong Sik Jang, Jung Yoon, Borae G. Park, Seong Jae Lee, Chae Seung Lim

**Affiliations:** 1 Department of Laboratory Medicine, College of Medicine, Korea University Guro Hospital, Korea University, Seoul 08308, Korea; plasmid18@hanmail.net (W.S.J.); yoteamoati@naver.com (J.Y.); borae.park@gmail.com (B.G.P.); 2 Department of Emergency Medicine, College of Medicine, Korea University Guro Hospital, Korea University, Seoul 08308, Korea; 3 Department of Medical Sciences, Graduate School of Medicine, Korea University, Seoul 02841, Korea; jhs878@naver.com; 4 Department of Polymer Engineering, The University of Suwon, Hwaseong, Gyeonggi 18323, Korea; sjlee@suwon.ac.kr

**Keywords:** sheathless, viscoelastic fluid, separation, shape, *Candida*

## Abstract

Rapid and accurate identification of *Candida albicans* from among other candida species is critical for cost-effective treatment and antifungal drug assays. Shape is a critical biomarker indicating cell type, cell cycle, and environmental conditions; however, most microfluidic techniques have been focused only on size-based particle/cell manipulation. This study demonstrates a sheathless shape-based separation of particles/cells using a viscoelastic non-Newtonian fluid. The size of *C. albicans* was measured at 37 °C depending on the incubation time (0 h, 1 h, and 2 h). The effects of flow rates on the flow patterns of candida cells with different shapes were examined. Finally, 2-h-incubated candida cells with germ tube formations (≥26 μm) were separated from spherical candida cells and shorter candida cells with a separation efficiency of 80.9% and a purity of 91.2% at 50 μL/min.

## 1. Introduction

*Candida albicans* (*C. albicans*) is known to be the most pathogenic among more than 150 species of the genus *Candida*: it can cause chronic infections in the bloodstream. Early diagnosis of *C. albicans* fungal infections and rapid identification of *C. albicans* from among other candida species are critical for targeted and cost-effective antifungal treatment of candida infections in the bloodstream [1]. *C. albicans* grow as budding yeast or in a filamentous hyphal form (short germ tubes): this is dependent on growth conditions. When *C. albicans* is incubated in human or rabbit serum at 37 °C for 2–3 h, short germ tubes are induced from the mother yeast cell [2]. Conventionally, a germ tube test (GTT) is used to identify *C. albicans*; however, the application of this technique is limited due to its time-consuming and labor-intensive process [3]. Therefore, a microfluidic technique to separate *C. albicans* with germ tube formation from spherical-shaped candida cells needs to be developed for the identification of *C. albicans*. In addition, germ tube formation in yeast is known to be associated with pathogenicity and mucosal invasiveness [4,5]. Therefore, in antifungal drug assays, to hinder germ tube formation, there is a need to isolate *C. albicans* with germ tubes [6,7].

Recent advancements in microfluidics have enabled an increased use of microfluidic techniques for the separation of particles/cells from a heterogeneous mixture sample in biological, chemical, and clinical applications [8,9,10]. Microfluidic separation techniques can be divided into two categories, i.e., active and passive methods, depending on the use of external force fields. Active methods adopt various external force fields, such as electric [11,12,13], magnetic [14,15], and acoustic fields [16,17,18,19,20], to separate particles/cells. Meanwhile, without the use of external forces, passive methods rely only on channel geometry and/or the hydrodynamic effects of fluid flow [21,22,23,24,25,26].

However, most current microfluidic-based techniques use size-based particle/cell separation, although shape can also be a critical biomarker of cell type and cell cycle [27,28,29,30,31]. A few microfluidic techniques have been used for shape-based separation, implementing dielectrophoresis [31], hydrodynamic filtration [32], deterministic lateral displacement [33], and inertial microfluidics [34]. However, dielectrophoresis requires an external setup and conductivity control over the cell medium, and the hydrodynamic filtration technique is limited by low throughput (~15 μL/min). Meanwhile, deterministic lateral displacement requires a complicated microstructure design.

Viscoelastic non-Newtonian microfluidics has gained much attention due to the intrinsic nonlinear elastic properties of viscoelastic fluids. In a non-Newtonian fluid, the nonuniform distribution of the first normal stress difference (*N*_1_) can drive suspended particles/cells laterally in a simple straight microchannel, eliminating the need for complicated channel structures [35,36,37]. Compared to other passive methods, the manipulation of viscoelastic particles/cells can be achieved over a wide working range of flow rates. Therefore, viscoelastic microfluidics has been applied in three-dimensional focusing [36,38,39] and size-based separation [37,40,41,42,43] of particles/cells. Of late, viscoelastic microfluidics has been utilized to separate spherical and peanut-shaped microparticles based on shape differences [44,45]. In addition, a viscoelastic sheathless shape-based separation technique has been applied to drug-treated human fungal pathogens using differences in morphology [46]. However, there is still room for improvement in device throughput and applications to various biological particles with different shapes [34,47].

More recently, our group demonstrated a microfluidic device for viscoelastic sheathless separation, which consisted of initialization of all the particles and continuous separation of particles through size-dependent lateral displacement [41,42,43]. Although this technique was utilized to manipulate biological samples, such as malaria parasites and blood components, it has not been applied in shape-dependent particle/cell separation.

In this study, we demonstrated sheathless separation based on shape differences using a viscoelastic fluid in a high-aspect ratio (*AR* = height/width) two-stage device. We examined the size and flow patterns of candida cells incubated for 0 h, 1 h, and 2 h at 37 °C, depending on the flow rates, during prefocusing of cells before the separation process. In addition, the flow characteristics of candida cells with different shapes were examined: finally, using the effect of shape-dependent elastic forces, the device was utilized for shape-based separation of rod-shaped candida cells from spherical candida cells with high purity at a flow rate of 50 μL/min for clinical diagnosis and pharmaceutical research [6,7]. To the best of the authors’ knowledge, this is the first report on the separation of candida cells based on germ tube formation using microfluidic techniques.

## 2. Working Principle

**Figure 1:**
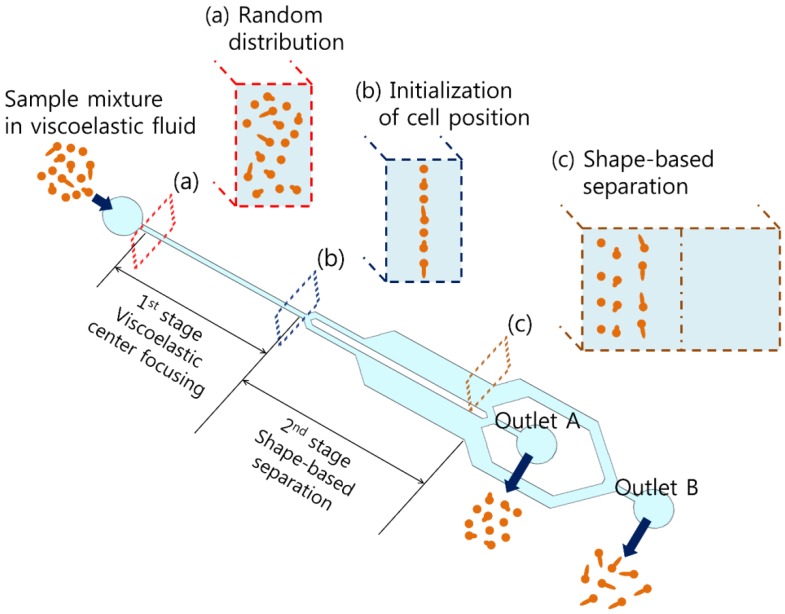
Schematic of sheathless shape-based separation of candida cells using a viscoelastic non-Newtonian fluid. A sample mixture containing candida cells with different shapes randomly distributed in viscoelastic fluid was injected through the inlet of a high-aspect ratio microchannel. Due to shape-dependent viscoelastic separation, rod-shaped candida cells are collected through the rear outlet (outlet B), whereas spherical candida cells are removed through the center outlet (outlet A).

A schematic of the device for the sheathless shape-based separation of cells using a viscoelastic non-Newtonian fluid is depicted in Figure 1. The device consists of a microfluidic channel of high *AR* with one inlet and two outlets. In addition, the microfluidic channel comprises two consecutive stages: the first stage is for the initialization of cell location and the second stage is for the separation of cells with different shapes. At the inlet, the initial sample (containing cells of different shapes in viscoelastic fluid) is injected. In pressure-driven flows of dilute polymer solutions, intrinsic nonlinear elastic force arises, which can be described in terms of the first and second normal stress difference, $N_{1}\left(\dot{\gamma }\right)=\sigma_{xx}-\sigma_{yy}$ and $N_{2}\left(\dot{\gamma }\right)=\sigma_{yy}-\sigma_{zz}$, respectively [48]. Here, $\sigma_{ii}$, *x*, *y*, and *z* are the diagonal component of the stress tensor, the direction of the flow, the direction of the velocity gradient, and the vorticity direction, respectively. In most cases in viscoelastic microfluidics, the $N_{2}$ contribution is neglected (*N*_1_ > 0, *N*_2_ ≈ 0). The elastic force (*F*_E_) exerted on the suspended cells is dependent on the particle size, based on the following relationship [36]:

(1) $$F_{E}\sim a^{3}\frac{\partial N_{1}}{\partial x}$$

Here, *x* is the lateral distance.

In the first stage, randomly distributed cells are focused on the channel center (where the first normal stress difference is minimal) [42], as the width of the first stage is designed to meet the condition under which the blockage ratio of spherical candida cells is larger than 0.1 (β=a/w, *a* = cell diameter, and *w* = channel width) [49], as shown in Figure 1a. At the bifurcation, the lateral position of the pre-aligned cells is initialized along the inner walls of the channel, as shown in Figure 1b. Next, in the second stage, all cells experience elastic force toward the channel center, which is dependent on the cell size, based on the following relationship [42,50,51]:

(2) $$\frac{V_{E}}{V_{m}}\sim Wi{\left(\frac{a}{h}\right)}^{2}\frac{\partial \dot{\gamma }}{\partial x}$$

Here, *V_E_* is the lateral migration velocity, *h* is the channel height, and $\dot{\gamma }$ is the localized shear rate. Rod-shaped candida cells incubated at 37 °C, have shown germ tubes induced from the mother yeast cell, which might increase the volume of the cell. During the flow, the axial direction of candida cells with germ tubes is mostly parallel to the flow direction. Due to the increased projection area of the cells, candida cells with germ tubes experience a larger wall repulsion force toward the center, which is perpendicular to the flow direction. The width of the second stage was designed to be able to focus the rod-shaped candida cells with germ tubes at the center. Consequently, candida cells with germ tubes migrate faster toward the center than spherical cells without germ tubes, and they can be separated using two different outlets (Figure 1c). Spherical cells are removed through the center outlet (outlet A), whereas rod-shaped cells are collected from the rear outlet (outlet B).

To characterize the fluid and cell dynamics during the viscoelastic fluid flow (where various forces, including inertia lift force and elastic force, simultaneously affect the flow characteristics of cells), nondimensional numbers, such as the Reynolds number (*Re*), Weissenberg number (*Wi*), and elasticity number (*El*), need to be adopted. The Reynolds number (*Re*) describes the ratio of inertial force to viscous force, whereas the Weissenberg number (*Wi*) describes the ratio of elastic force to viscous force, as follows:

(3) Re=ρVmDhηc

(4) $$\mathrm{Wi}=\lambda {\dot{\gamma }}_{c}$$

Here, *ρ, V_m_, D_h_, η_c_, λ,* and $\dot{\gamma_{c}}$ represent the solution density, mean flow velocity, hydraulic diameter, characteristic viscosity of the solution, fluid relaxation time, and characteristic shear rate, respectively. The elasticity number (*El = Wi/Re*) is the ratio of the elastic force to inertial force.

## 3. Materials and Methods

### 3.1. Device Design and Fabrication

A polydimethylsiloxane (PDMS) microfluidic channel was fabricated using a standard soft lithography technique with a replica mold, which was fabricated using SU-8 negative photoresistor (MicroChem, Newton, MA, USA) on a silicon wafer. The PDMS base and curing agent (Sylgard 184, Dow Corning, Midland, MI, USA) were mixed at a 10:1 ratio and cast over the replica mold. The mixture was degassed in a vacuum chamber and thermally cured for 1 h at 80 °C. The cured PDMS channel mold was peeled off the mold, cut, and bonded on a glass slide with oxygen plasma treatment (CUTE, Femto Science, Gyeonggi-do, South Korea). The widths of the first and the second stage were designed to focus small, spherical candida cells and rod-shaped candida cells with germ tubes, respectively, on the center plane. The width of the germ tube is known as half of the width of the mother yeast cell. The volume of the cells was calculated by measuring the average length of the 3-h-incubated candida cells (~90 μm). Assuming a sphere of the same volume as the calculated volume of candida cells, the diameter was calculated and the width of the second-stage channel was designed to have a blockage ratio larger than 0.1. Therefore, the fabricated PDMS microfluidic channel has a high *AR* rectangular cross-section with a width of 40 μm in the first stage and 70 μm in the second stage and a height of 120 μm. The lengths of both stages were designed as 3 cm. The expansion region was designed with a width of 630 μm to maximize the differences in the lateral migration displacements in the second stage [42]. In addition, the expansion region was used to facilitate evaluating the lateral migration displacements of the cells, since the second stage was too narrow to analyze the cell distribution. To minimize unwanted hydrophobic interactions between particles and channel surfaces, the PDMS microfluidic channel was treated with Tween 20 [52].

### 3.2. Sample Preparation

As a non-Newtonian fluid, hyaluronic acid (HA) sodium salt (357 kDa, Lifecore Biomedical, Chaska, MN, USA) in phosphate-buffered saline (PBS) was prepared at a concentration of 0.1% (w/v). The high shear viscosity (*η_∞_*) of 0.1% HA solution has been reported to be 0.89 mPa·s, and the relaxation time has been reported to be 0.25 ms [49,53].

*C. albicans* SC5413 were provided by Dr. Jeong-Yoon Kim in the Department of Microbiology & Molecular Biology, Chungnam University, Korea. Yeasts were cultured overnight at 30 °C in 10 mL of yeast extract–peptone–dextrose (YPD) broth (Qbiogene). The cultured cells were quantified under phase-contrast microscopy (40× power) using a counting grid. To obtain candida cells with germ tubes grown from mother yeast cells, the standard protocol of a germ tube test was adopted [3,54]. For germ tubes with various lengths, the cultured candida cells were incubated in human serum at 37 °C for 1 h, 2 h, and 3 h. After incubation process, incubated candida cells were suspended in 0.1% HA solution and the cell suspension was injected into the microchannel at room temperature (~24 °C). During the experiment, there are no more morphological changes in *C. albicans* since the temperature and the suspending medium do not meet the conditions for germ tube formation.

### 3.3. Experimental Procedure

The sample solution was injected into a microchannel using a syringe pump (LSP01-1A, Longer Precision Pump Co., Ltd., Hebei, China). The particles/cells flowing in the microchannel were monitored using an inverted microscope (CKX41, Olympus, Tokyo, Japan) with a high-speed camera (V611, Phantom, Wayne, NJ, USA).

## 4. Results and Discussion

**Figure 2:**
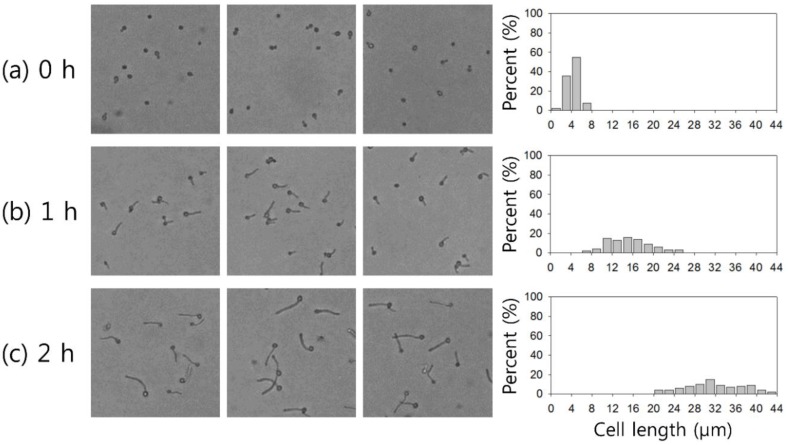
Size distribution of candida cells depending on incubation time: (**a**) 0 h, (**b**) 1 h, and (**c**) 2 h at 37 °C.

When candida cells are grown in human serum at 37 °C, germ tubes are formed, which are short-outgrowth, non-septate-germinating hyphae. Conventionally, *C. albicans* can be identified using a germ tube test (GTT) conducted on a subcultured colony [3]. As shown in Figure 2, the length of the germ tubes induced by 37 °C incubation is dependent on the incubation time: 4.38 ± 1.09 μm, 15.43 ± 4.13 μm, and 31.81 ± 5.58 μm after 0 h, 1 h, and 2 h of incubation, respectively.

To determine the flow rate conditions for candida cells at the center in the first stage, the flow patterns of particles with different shapes were monitored at the first bifurcation, i.e., at 3 cm downstream from the inlet, with flow rates ranging from 5 μL/min (*Re* = 1.15, *Wi* = 0.22, *El* = 0.19) to 200 μL/min (*Re* = 46.2, *Wi* = 9.02, *El* = 0.19). The size of *C. albicans* without 37 °C incubation was measured at 4.38 ± 1.09 μm, in which the blockage ratio (*β*) met the condition of being *β* ≥ 0.1 for the prefocusing of cells.

**Figure 3:**
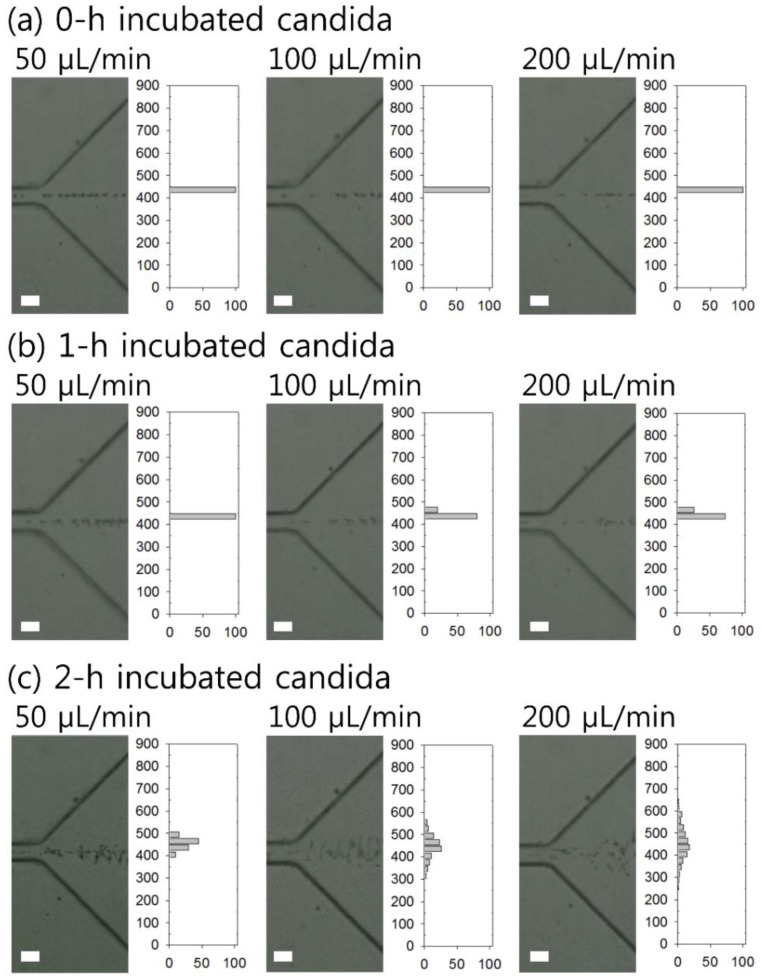
Flow rate-dependent focusing characteristics and lateral distribution of *Candida albicans* incubated for (**a**) 0 h, (**b**) 1 h, and (**c**) 2 h at 37 °C at flow rates of 50 μL/min, 100 μL/min, and 200 μL/min, respectively. The scale bar is 50 μm.

Figure 3 shows the stacked microscopic images of flow rate-dependent characteristics of cells and the lateral distribution of candida cells suspended in 0.1% HA solution at flow rates of 50 μL/min, 100 μL/min, 200 μL/min, and 300 μL/min, depending on the incubation time (0 h, 1 h, and 2 h). Before the use of our two-stage device, a microfluidic channel containing a rectangular straight channel and an expansion region instead of the first bifurcation was constructed to examine the distribution of particles. The straight channel was designed to have the same dimensions as the first-stage channel (width 40 μm, height 120 μm, and length 3 cm), and the width of the expansion region was 900 μm to enable the observation of focused particles using a high-speed camera. The 900-μm-wide expansion region was divided into 30 virtual segments. The number of cells in each segment of the 30-μm-wide expansion region was normalized by the number of total cells flowing in the entire width. The lateral position of cells with germ tubes was determined based on the geometrical center of the cell.

At a flow rate lower than 10 μL/min (*Re* = 2.31, *Wi* = 0.45, *El* = 0.19), cells could not be center-focused: they were randomly distributed due to low elastic force (data not shown). As the flow rate increased, the nondimensional numbers *Re* and *Wi* also increased, resulting in enhanced inertial lift force and elastic force. At *Q* ≥ 25 μL/min (*Re* = 5.78, *Wi* = 1.12, *El* = 0.19), all of the candida cells with incubation times of 0 h, 1 h, and 2 h were successfully focused in a single band and remained focused at flow rates up to 50 μL/min (*Re* = 11.5, *Wi* = 2.25, *El* = 0.19), as shown in Figure 2a–c. Without incubation at 37 °C (0-h sample in Figure 2a and 1-h-incubated candida cells (Figure 2b)), the candida cells were tightly focused at flow rates of 25 ≤ *Q* < 200 μL/min. However, once the flow rate increased to 100 μL/min (*Re* = 23.1, *Wi* = 4.51, *El* = 0.19), the focused state of the candida cells incubated for 2 h slightly dispersed, which might have been due to the longer length of the cells. In addition, due to the inertial effect at high flow rates, particles began to migrate toward the equilibrium positions along the sidewalls [55]. Therefore, the flow rate was chosen as 25 ≤ *Q* < 100 μL/min to evaluate the viscoelastic lateral migration depending on the shape of the candida cells.

To evaluate the viscoelastic lateral migration based on the length of the germ tubes, *C. albicans* were prepared through incubation for 0 h, 1 h, and 2 h. Three-hour-incubated *C. albicans* were excluded from the evaluation. Although cells were focused along the centerline in the first stage, approximately 72% of 3-h-incubated *C. albicans* could not be initialized at the first bifurcation due to their extreme length and asymmetry. The asymmetrical coefficient (α) of *C. albicans* was defined as the angle between a straight line connecting two points located at each end of the candida cells and a tangent line at the end of the germ tube, divided by 90° (see Supplementary Materials, Figure S1). For candida cells incubated for 3 h, many of the cells had a curved shape; moreover, ~40% of the candida cells had an asymmetrical coefficient higher than 0.3. Therefore, noninitialized 3-h-incubated *C. albicans* were randomly distributed across the channel in the expansion region, even beyond the centerline of the microchannel (see Supplementary Materials, Figure S2). Although an asymmetrical coefficient was adopted to evaluate the curved shape of candida cells in this study, it is worth examining the effect of asymmetry of candida cells on flow characteristics through analytical and experimental studies (as a potential future work) [56].

**Figure 4:**
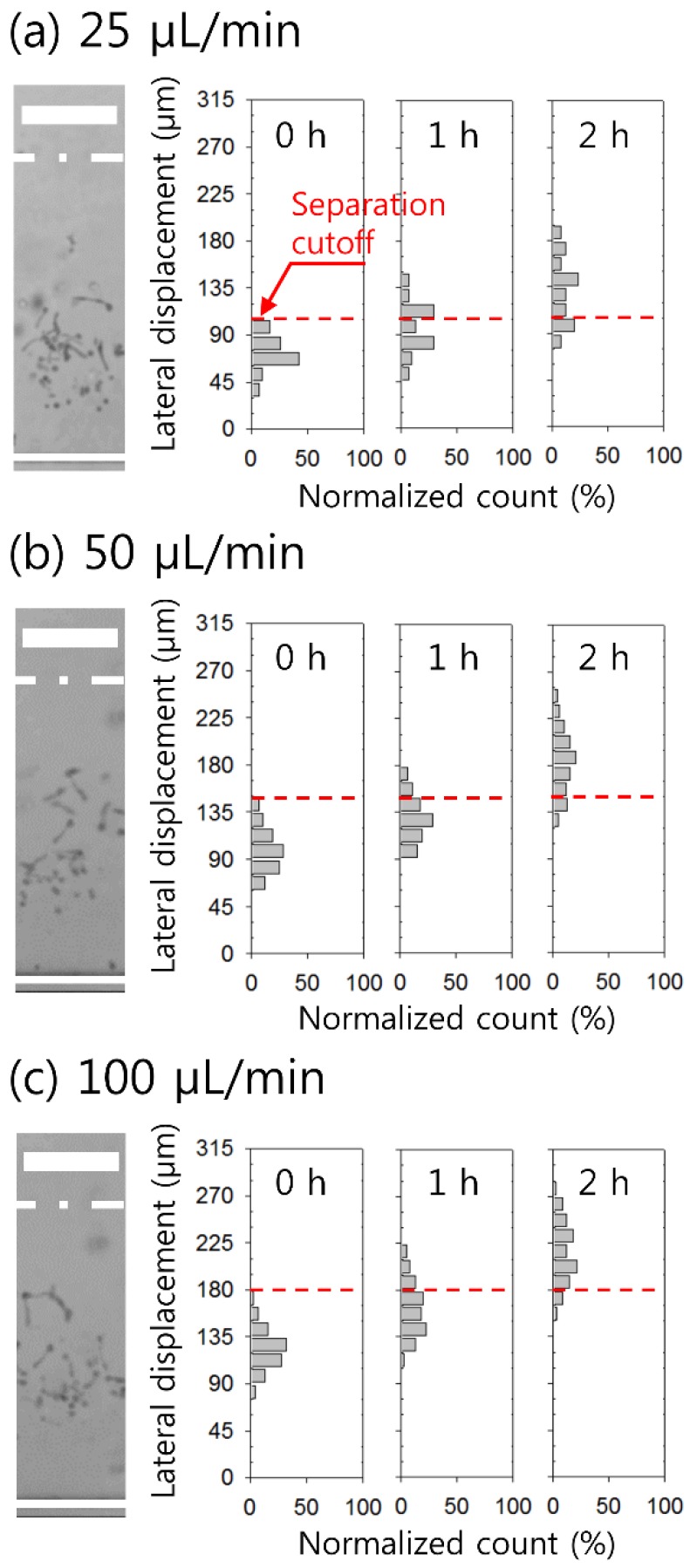
Effect of flow rates on viscoelastic lateral migration of candida cells incubated at 37 °C. Stacked images of cells in the expansion region and cell distribution depending on incubation time (0 h, 1 h, and 2 h) are shown at flow rates of (**a**) 25 μL/min, (**b**) 50 μL/min, and (**c**) 100 μL/min. Red dotted lines in (a–c) show the separation cutoff. The scale bar is 100 μm.

The flow characteristics of the incubated candida cells suspended in 0.1% HA solution at a concentration of ~1 × 10^4^ cells/mL were examined at different flow rates of 25 μL/min, 50 μL/min, and 100 μL/min. For drug assays or antifungal susceptibility testing, candida cells at a concentration ranging from 1 × 10^3^ to 5 × 10^8^ cells/ml are mainly used [6,57,58,59,60]. In this study, we decided to use candida cells at ~1 × 10^4^ cells/mL, which was within the corresponding concentration range. Figure 4 shows the stacked microscopic images and lateral distribution of candida cells incubated at 37 °C for 0 h, 1 h, and 2 h in the expansion region at different flow rates (25 μL/min, 50 μL/min, and 100 μL/min). The red dotted lines in Figure 4a–c show the separation cutoff to separate the 1-h- and 2-h-incubated *C. albicans* samples from the others at each flow rate. Upon the incubation of *C. albicans* in human or rabbit serum at 37 °C for 2–3 h, the growth of short germ tubes is induced: these have half the width and 3–4 times the length of the mother yeast cell [2]. As the incubation time at 37 °C increases, the length of the germ tube increases, thereby increasing the hydraulic diameter of the candida cell due to the increased cell volume. Therefore, based on the cell diameter dependence of the elastic force ($F_{E}\propto a^{3}$, where *a* is the hydraulic diameter of the cell), *C. albicans* experiences higher elastic forces and exhibits greater lateral migration.

As shown in Figure 4a, at *Q* = 25 μL/min (*Re* = 5.78, *Wi* = 1.12, *El* = 0.19), candida cells without 37 °C incubation (0-h sample) exhibited lateral migration of 72.82 ± 15.96 μm, but 1-h- and 2-h-incubated candida cells showed lateral displacement of 96.53 ± 27.58 μm and 132.11 ± 31.06 μm, respectively. Thus, the streams partially overlapped with each other, making the separation incomplete. For the 1-h-incubated sample, the red dotted line located 105 μm away from the inner wall (*y* = 0) showed the separation cutoff, enabling the removal of the 0-h-incubated sample and the separation of the 1-h sample at a separation efficiency of 41.9%. For the 2-h-incubated sample, the separation efficiency was 73.0% based on a separation cutoff at *y* = 105 μm.

At *Q* = 50 μL/min (*Re* = 11.5, *Wi* = 2.25, *El* = 0.19) (Figure 4b), candida cells incubated at 37 °C for 0 h, 1 h, and 2 h migrated laterally toward the center by 98.96 ± 20.66 μm, 128.8 ± 21.50 μm, and 183.3 ± 30.85 μm, respectively. Based on the separation cutoff located at *y* = 150 μm, the separation efficiencies were 17.67% for the 1-h-incubated sample and 82.03% for the 2-h-incubated sample. As shown in Figure 4c, the increase in flow rate to 100 μL/min (*Re* = 23.1, *Wi* = 4.51, *El* = 0.19) appeared to diminish the difference in the lateral displacement of candida cells. Lateral displacements of candida cells were 122.71 ± 19.35 μm for the 0-h sample, 161.81 ± 25.83 μm for the 1-h-incubated sample, and 215.47 ± 29.28 μm for the 2-h-incubated sample, which showed a largely overlapped distribution. Using the separation cutoff of 180 μm away from the inner wall, the separation efficiencies were 25% for the 1-h-incubated sample and 88.2% for the 2-h-incubated sample. Therefore, in our experimental setup, based on the difference between lateral displacements, the flow rate was determined at 50 μL/min for the separation of candida cells with different lengths.

**Figure 5:**
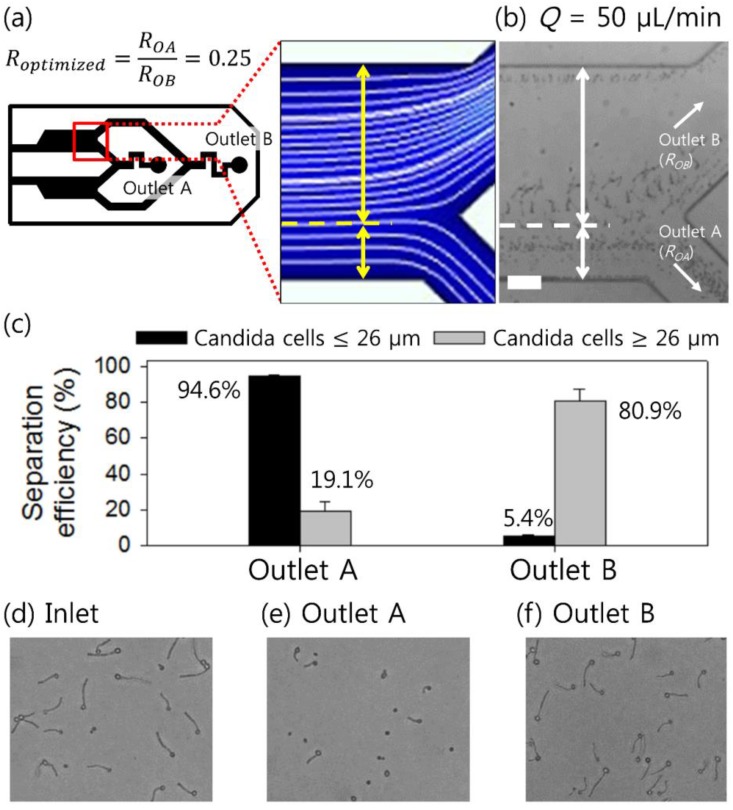
Separation of candida cells depending on germ tube formation. (**a**) Schematic image of the device outlet with a fluidic resistance ratio of *R_OA_*/*R_OB_* = 1:4 and a numerical simulation of the flow streamline. (**b**) Separation of candida cells incubated at 37 °C for 0 h and 2 h at *Q* = 50 μL/min. The scale bar is 100 μm. (**c**) The separation efficiency of candida cells larger than 26 μm at each outlet for the collected sample. Microscopic images show (**d**) the sample injected into the inlet and the collected cells at (**e**) outlet A and (**f**) outlet B.

To evaluate the clinical applicability of the device, shape-based separation of candida cells depending on germ tube length was performed using the optimized experimental conditions. On the basis of the separation cutoff (*y* = 150 μm) determined by the cell distribution at 50 μL/min (in Figure 4b), an optimized device was designed to have a fluidic resistance ratio of *R_OA_*/*R_OB_* = 1:4 [26,61], the schematic of which is shown in Figure 5a. Here, *R_OA_* and *R_OB_* are the fluidic resistances at outlet A and outlet B, respectively.

To determine the widths of the outlet bifurcation channel, we conducted a numerical simulation using the Oldroyd-B model in a straight microchannel. For the simulation, the momentum equation is expressed as

(5) Re(u·∇)u=∇·(−pI+(ηs/η)[∇u+(∇u)T]+T.

In addition, the extra stress contribution due to viscoelasticity is defined as

(6) $$T+\mathrm{Wi}\overset{‘}{T}=\left(\eta_{p}/\eta \right){\left[\nabla u+{\left(\nabla u\right)}^{T}\right]}_{\nabla },$$

where $\overset{‘}{T}=\frac{\partial T}{\partial t}+\left(u\cdot \nabla \right)T-\left[\left(\nabla u\right)T+T{\left(\nabla u\right)}^{T}\right]$. These governing equations are nondimensionalized by *Re_c_* and *Wi*. Here, *λ* is the characteristic relaxation time, *η_s_* is the relative solvent viscosity, *η_p_* is the relative polymer viscosity, and *η* is the total viscosity (*η* = *η_s_* + *η_p_.*). The numerical simulation results for flow streamlines at the outlet bifurcation are shown in Figure 5a.

Figure 5b shows stacked microscopic images over time at the second bifurcation of the microchannel during the separation process, which was recorded using a high-speed camera (see Supplementary Materials, Video S1). A mixture of 0-h- and 2-h-incubated candida cells was injected at the inlet at *Q* = 50 μL/min and randomly distributed. Candida cells longer than ~26 μm among the 2-h-incubated candida cells were separated at the side of the outlet (outlet B), whereas all other cells (≤26 μm) flowing under the separation threshold were removed through outlet A. Using the cells collected at outlets A and B, the shape-based separation performance was evaluated. As shown in Figure 5c, 94.6% of candida cells shorter than 26 μm were removed through outlet A, whereas 80.9% of candida cells longer than 26 μm were collected at outlet B with a purity of 91.2%. Purity is defined as the ratio of the number of target particles/cells at the target outlet to the total number of particles/cells found at the target outlet. The separation efficiency and purity of the shape-based separation of *C. albicans* were reduced compared to the size-based separation (~94% of separation efficiency and ~99% of purity) [42]: this may have been due to the asymmetry of *C. albicans*. In Figure 5b, few cells are seen near the top wall, which seems to be an error in the experiments and did not significantly affect the experimental results. This additional stream of cells near the top wall might have occurred due to trapped cells at the corner or aggregated cells. Once the cells are trapped in the corners of a rectangular channel, cells are entrained along the corners due to the nonuniform normal stress gradient [62,63,64]. Meanwhile, in biological samples, the aggregation tendency of cells may induce unexpected, undesirable effects on experimental results [65,66]. In our experiments, candida cells were at a low concentration (~1 × 10^4^ cells/mL), which might rarely cause cell aggregation-related problems. However, cell aggregation may have affected the experimental results with increased cell concentration, so further studies are required to optimize the cell concentration for our devices. Meanwhile, a small number of candida cells with germ tubes longer than 26 μm were observed to flow with spherical candida cells and shorter candida cells near the inner wall of the expansion region. Candida cells flowing along the inner walls at the first bifurcation were affected by the strong corner-directed elastic force and trapped in the microchannel corners, which affected the separation efficiency. Figure 5d–f shows microscopic images of the sample for injection and the collected samples at outlets A and B, respectively.

In previous reports on shape-based particle separation using viscoelastic fluid, the peanut-shaped particles underwent an in-plane rotation, which affected their flow characteristics [44]. However, in our device, the rotation of candida cells with germ tube formation was not observed. In further studies, it is worth examining flow characteristics based on rotational motion by modulating the polymer concentration and flow conditions.

A polymer solution (HA solution) with low viscosity but high elasticity was employed [49,53]. The device throughput can be further enhanced by modulating the viscoelasticity and flow resistance of the polymer solution. Higher viscoelasticity can be achieved by increasing the concentration of the polymer solution; moreover, flow resistance can be reduced by shortening the channel length or multiplexing the devices [53,67].

With regard to shape-based separation of candida cells using our device, it was confirmed that separation efficiency is affected by asymmetry due to the shape of the formed germ tube. In other studies, the device enables the separation of cells based on shape differences without curvature, which can be a useful biomarker indicating cell type, cell cycle, and differences based on environmental conditions (e.g., fungal pathogens, microalgae, and rod-shaped bacteria (e.g., bacilli)) [34,46,47,68]. In addition, additional numerical analyses and simulations are required to calculate the elastic force exerted on nonspherical particles/cells and to predict the flow characteristics of those in the viscoelastic fluid. In further numerical studies, three-dimensional modeling should be used to accurately predict the flow stream split at the trifurcation outlet due to the nonuniform distribution of fluidic velocity across the channel height. Meanwhile, by considering the blockage ratio and the viscoelasticity of the polymer solution, our device can be applied in the separation of submicron-sized particles.

## 5. Conclusions

In summary, we described a shape-based *C. albicans* separation device consisting of two stages for cell focusing and separation using a viscoelastic fluid. The sizes of the candida cells were measured depending on the incubation time at 37 °C. The flow rate-dependent focusing characteristics of the cells were examined at 25 ≤ *Q* ≤ 300 μL/min. At 50 μL/min, all candida cells incubated for 0 h, 1 h, and 2 h at 37 °C were center-focused in the first-stage microchannel. On the basis of the increase in cell size with the incubation time, lateral migration of the candida cells was also examined. Finally, 2-h-incubated candida cells could be separated with 80.9% separation efficiency and 91.2% purity at optimal conditions (*El* = 0.19, *Wi* = 2.25, asymmetrical coefficient ≤0.3, and *R_OA_*/*R_OB_* = 1:4). Our device enables the continuous, sheathless, shape-based separation of cells; thus, it can be a powerful tool for separation, using shape differences as a biomarker.

## Supplementary Materials

The following are available online at https://www.mdpi.com/2072-666X/10/12/817/s1, Figure S1: Asymmetrical coefficient (α) of 3-h-incubated candida cells: (a) α = 0, (b) α = 0.25, and (c) α = 0.61; Figure S2: Flow characteristics of candida cells incubated for 3 h at 37 °C (a) at the first bifurcation and (b) at the second bifurcation at *Q* = 50 μL/min. Video S1: Separation of candida cells incubated at 37 °C for 0 h and 2 h at *Q* = 50 μL/min.

## References

[1] Pappas P.G., Rex J.H., Sobel J.D., Filler S.G., Dismukes W.E., Walsh T.J., Edwards J.E. (2004). Guidelines for treatment of candidiasis. Clin. Infect. Dis..

[2] Mackenzie D. (1962). Serum tube identification of Candida albicans. J. Clin. Pathol..

[3] Sheppard D.C., Locas M.-C., Restieri C., Laverdiere M. (2008). Utility of the germ tube test for direct identification of Candida albicans from positive blood culture bottles. J. Clin. Microbiol..

[4] Braude A.I., Davis C.E., Fierer J., Philadelphia W.B. (1986). Infectious Diseases and Medical Microbiology.

[5] JO I., Eghubare A., Omoregie R. (2005). Germ Tube Formation in Candida Albicans: Evaluation of Human and Animal Sera and Incubation Atmosphere. Shiraz E-Medical J..

[6] Brayman T.G., Wilks J.W. (2003). Sensitive assay for antifungal activity of glucan synthase inhibitors that uses germ tube formation in Candida albicans as an end point. Antimicrob. Agents Chemother..

[7] Rusu E., Radu-Popescu M., Pelinescu D., Vassu T. (2014). Treatment with some anti-inflammatory drugs reduces germ tube formation in Candida albicans strains. Braz. J. Microbiol..

[8] Karimi A., Yazdi S., Ardekani A. (2013). Hydrodynamic mechanisms of cell and particle trapping in microfluidics. Biomicrofluidics.

[9] Pamme N. (2007). Continuous flow separations in microfluidic devices. Lab A Chip.

[10] Sajeesh P., Sen A.K. (2014). Particle separation and sorting in microfluidic devices: A review. Microfluid. Nanofluidics.

[11] Gascoyne P.R., Noshari J., Anderson T.J., Becker F.F. (2009). Isolation of rare cells from cell mixtures by dielectrophoresis. Electrophoresis.

[12] Pethig R. (2010). Dielectrophoresis: Status of the theory, technology, and applications. Biomicrofluidics.

[13] Regtmeier J., Duong T.T., Eichhorn R., Anselmetti D., Ros A. (2007). Dielectrophoretic manipulation of DNA: Separation and polarizability. Anal. Chem..

[14] Hejazian M., Li W., Nguyen N.-T. (2015). Lab on a chip for continuous-flow magnetic cell separation. Lab A Chip.

[15] Zhao W., Cheng R., Miller J.R., Mao L. (2016). Label-free microfluidic manipulation of particles and cells in magnetic liquids. Adv. Funct. Mater..

[16] Augustsson P., Magnusson C., Nordin M., Lilja H., Laurell T. (2012). Microfluidic, label-free enrichment of prostate cancer cells in blood based on acoustophoresis. Anal. Chem..

[17] Laurell T., Petersson F., Nilsson A. (2007). Chip integrated strategies for acoustic separation and manipulation of cells and particles. Chem. Soc. Rev..

[18] Li P., Mao Z., Peng Z., Zhou L., Chen Y., Huang P.-H., Truica C.I., Drabick J.J., El-Deiry W.S., Dao M. (2015). Acoustic separation of circulating tumor cells. Proc. Natl. Acad. Sci. USA.

[19] Nam J., Lim H., Kim D., Shin S. (2011). Separation of platelets from whole blood using standing surface acoustic waves in a microchannel. Lab A Chip.

[20] Wang Z., Zhe J. (2011). Recent advances in particle and droplet manipulation for lab-on-a-chip devices based on surface acoustic waves. Lab A Chip.

[21] Martel J.M., Toner M. (2014). Inertial focusing in microfluidics. Annu. Rev. Biomed. Eng..

[22] McGrath J., Jimenez M., Bridle H. (2014). Deterministic lateral displacement for particle separation: A review. Lab A Chip.

[23] Warkiani M.E., Khoo B.L., Wu L., Tay A.K.P., Bhagat A.A.S., Han J., Lim C.T. (2016). Ultra-fast, label-free isolation of circulating tumor cells from blood using spiral microfluidics. Nat. Protoc..

[24] Zhou J., Tu C., Liang Y., Huang B., Fang Y., Liang X., Papautsky I., Ye X. (2018). Isolation of cells from whole blood using shear-induced diffusion. Sci. Rep..

[25] Zhou J., Papautsky I. (2019). Size-dependent enrichment of leukocytes from undiluted whole blood using shear-induced diffusion. Lab A Chip.

[26] Takagi J., Yamada M., Yasuda M., Seki M. (2005). Continuous particle separation in a microchannel having asymmetrically arranged multiple branches. Lab A Chip.

[27] Ebert E.C., Nagar M., Hagspiel K.D. (2010). Gastrointestinal and hepatic complications of sickle cell disease. Clin. Gastroenterol. Hepatol..

[28] Janča J., Halabalová V., Růžička J. (2010). Role of the shape of various bacteria in their separation by microthermal field-flow fractionation. J. Chromatogr. A.

[29] Martin S.G. (2009). Geometric control of the cell cycle. Cell Cycle.

[30] Mitragotri S., Lahann J. (2009). Physical approaches to biomaterial design. Nat. Mater..

[31] Valero A., Braschler T., Rauch A., Demierre N., Barral Y., Renaud P. (2011). Tracking and synchronization of the yeast cell cycle using dielectrophoretic opacity. Lab A Chip.

[32] Sugaya S., Yamada M., Seki M. (2011). Observation of nonspherical particle behaviors for continuous shape-based separation using hydrodynamic filtration. Biomicrofluidics.

[33] Beech J.P., Holm S.H., Adolfsson K., Tegenfeldt J.O. (2012). Sorting cells by size, shape and deformability. Lab A Chip.

[34] Li M., Muñoz H.E., Goda K., Di Carlo D. (2017). Shape-based separation of microalga Euglena gracilis using inertial microfluidics. Sci. Rep..

[35] d’Avino G., Maffettone P., Greco F., Hulsen M. (2010). Viscoelasticity-induced migration of a rigid sphere in confined shear flow. J. Non-Newton. Fluid Mech..

[36] Leshansky A.M., Bransky A., Korin N., Dinnar U. (2007). Tunable nonlinear viscoelastic “focusing” in a microfluidic device. Phys. Rev. Lett..

[37] Nam J., Lim H., Kim D., Jung H., Shin S. (2012). Continuous separation of microparticles in a microfluidic channel via the elasto-inertial effect of non-Newtonian fluid. Lab A Chip.

[38] Ahn S.W., Lee S.S., Lee S.J., Kim J.M. (2015). Microfluidic particle separator utilizing sheathless elasto-inertial focusing. Chem. Eng. Sci..

[39] Nam J., Jang W.S., Lim C.S. (2019). Non-electrical powered continuous cell concentration for enumeration of residual white blood cells in WBC-depleted blood using a viscoelastic fluid. Talanta.

[40] Liu C., Guo J., Tian F., Yang N., Yan F., Ding Y., Wei J., Hu G., Nie G., Sun J. (2017). Field-free isolation of exosomes from extracellular vesicles by microfluidic viscoelastic flows. ACS Nano.

[41] Nam J., Namgung B., Lim C.T., Bae J.-E., Leo H.L., Cho K.S., Kim S. (2015). Microfluidic device for sheathless particle focusing and separation using a viscoelastic fluid. J. Chromatogr. A.

[42] Nam J., Shin Y., Tan J.K.S., Lim Y.B., Lim C.T., Kim S. (2016). High-throughput malaria parasite separation using a viscoelastic fluid for ultrasensitive PCR detection. Lab A Chip.

[43] Nam J., Tan J.K.S., Khoo B.L., Namgung B., Leo H.L., Lim C.T., Kim S. (2015). Hybrid capillary-inserted microfluidic device for sheathless particle focusing and separation in viscoelastic flow. Biomicrofluidics.

[44] Lu X., Xuan X. (2015). Elasto-inertial pinched flow fractionation for continuous shape-based particle separation. Anal. Chem..

[45] Lu X., Zhu L., Hua R.-M., Xuan X. (2015). Continuous sheath-free separation of particles by shape in viscoelastic fluids. Appl. Phys. Lett..

[46] Li D., Zielinski J., Kozubowski L., Xuan X. (2018). Continuous sheath-free separation of drug-treated human fungal pathogen Cryptococcus neoformans by morphology in biocompatible polymer solutions. Electrophoresis.

[47] Schaap A., Dumon J., Den Toonder J. (2016). Sorting algal cells by morphology in spiral microchannels using inertial microfluidics. Microfluid. Nanofluidics.

[48] Barnes H.A., Hutton J.F., Walters K. (1989). An Introduction to Rheology.

[49] Lim E.J., Ober T.J., Edd J.F., Desai S.P., Neal D., Bong K.W., Doyle P.S., McKinley G.H., Toner M. (2014). Inertio-elastic focusing of bioparticles in microchannels at high throughput. Nat. Commun..

[50] Seo K.W., Byeon H.J., Huh H.K., Lee S.J. (2014). Particle migration and single-line particle focusing in microscale pipe flow of viscoelastic fluids. RSC Adv..

[51] Tehrani M. (1996). An experimental study of particle migration in pipe flow of viscoelastic fluids. J. Rheol..

[52] Zhou Y., Basu S., Wohlfahrt K.J., Lee S.F., Klenerman D., Laue E.D., Seshia A.A. (2016). A microfluidic platform for trapping, releasing and super-resolution imaging of single cells. Sens. Actuators B Chem..

[53] Lim H., Back S.M., Hwang M.H., Lee D.-H., Choi H., Nam J. (2019). Sheathless high-throughput circulating tumor cell separation using viscoelastic non-newtonian fluid. Micromachines.

[54] Matare T., Nziramasanga P., Gwanzura L., Robertson V. (2017). Experimental germ tube induction in Candida albicans: An evaluation of the effect of sodium bicarbonate on morphogenesis and comparison with pooled human serum. Biomed Res. Int..

[55] Zhou J., Giridhar P.V., Kasper S., Papautsky I. (2013). Modulation of aspect ratio for complete separation in an inertial microfluidic channel. Lab A Chip.

[56] Schuech R., Hoehfurtner T., Smith D.J., Humphries S. (2019). Motile curved bacteria are Pareto-optimal. Proc. Natl. Acad. Sci..

[57] Perumal P., Mekals S., Chaffin W.L. (2007). Role for cell density in antifungal drug resistance in candida albicans biofilms. Antimicrob. Agents Chemother..

[58] Seneviratne C.J., Jin L.J., Samaranayake Y.H., Samaranayake L.P. (2008). Cell density and cell aging as factors modulating antifungal resistance of candida albicans biofilms. Antimicrob. Agents Chemother..

[59] Li Y., Chang W., Zhang M., Li X., Jiao Y., Lou H. (2015). Synergistic and drug-resistant reversing effects of diorcinol D combined with fluconazole against Candida albicans. FEMS Yeast Res..

[60] De Luca C., Guglielminetti M., Ferrario A., Calabr M., Casari E. (2012). Candidemia: Species involved, virulence factors and antimycotic susceptibility. New Microbiol..

[61] Tu C., Zhou J., Liang Y., Huang B., Fang Y., Liang X., Ye X. (2017). A flexible cell concentrator using inertial focusing. Biomed. Microdevices.

[62] Seo K.W., Kang Y.J., Lee S.J. (2014). Lateral migration and focusing of microspheres in a microchannel flow of viscoelastic fluids. Phys. Fluids.

[63] D’Avino G., Greco F., Maffettone P.L. (2017). Particle migration due to viscoelasticity of the suspending liquid and its relevance in microfluidic devices. Annu. Rev. Fluid Mech..

[64] D’Avino G., Romeo G., Villone M.M., Greco F., Netti P.A., Maffettone P.L. (2012). Single line particle focusing induced by viscoelasticity of the suspending liquid: Theory, experiments and simulations to design a micropipe flow-focuser. Lab A Chip.

[65] Nam J., Lim H., Kim C., Kang J.Y., Shin S. (2012). Density-dependent separation of encapsulated cells in a microfluidic channel by using a standing surface acoustic wave. Biomicrofluidics.

[66] Murua A., Portero A., Orive G., Hernández R.M., de Castro M., Pedraz J.L. (2008). Cell microencapsulation technology: Towards clinical application. J. Control. Release.

[67] Warkiani M.E., Khoo B.L., Tan D.S.-W., Bhagat A.A.S., Lim W.-T., Yap Y.S., Lee S.C., Soo R.A., Han J., Lim C.T. (2014). An ultra-high-throughput spiral microfluidic biochip for the enrichment of circulating tumor cells. Analyst.

[68] Yuan D., Zhao Q., Yan S., Tang S.-Y., Zhang Y., Yun G., Nguyen N.-T., Zhang J., Li M., Li W. (2019). Sheathless separation of microalgae from bacteria using a simple straight channel based on viscoelastic microfluidics. Lab A Chip.

