# Supplementary material for: Sheathless Shape-Based Separation of Candida Albicans Using a Viscoelastic Non-Newtonian Fluid
Source: Micromachines (Basel). 2019 Nov 26;10(12):817. doi: 10.3390/mi10120817 (PMC6952941; doi:10.3390/mi10120817)
Supplement: Supplementary file 1 [file micromachines-10-00817-s001.zip › micromachines-644320-supplementary for proofreading/micromachines-644320-supplementary for proofreading.docx]

Sheathless Shape-Based Separation of *Candida Albicans* Using a Viscoelastic Non-Newtonian Fluid

Jeonghun Nam, Hyunseul Jee, Woong Sik Jang, Jung Yoon, Borae G. Park, Seong Jae Lee and Chae Seung Lim

1. Asymmetry of *Candida* Cells


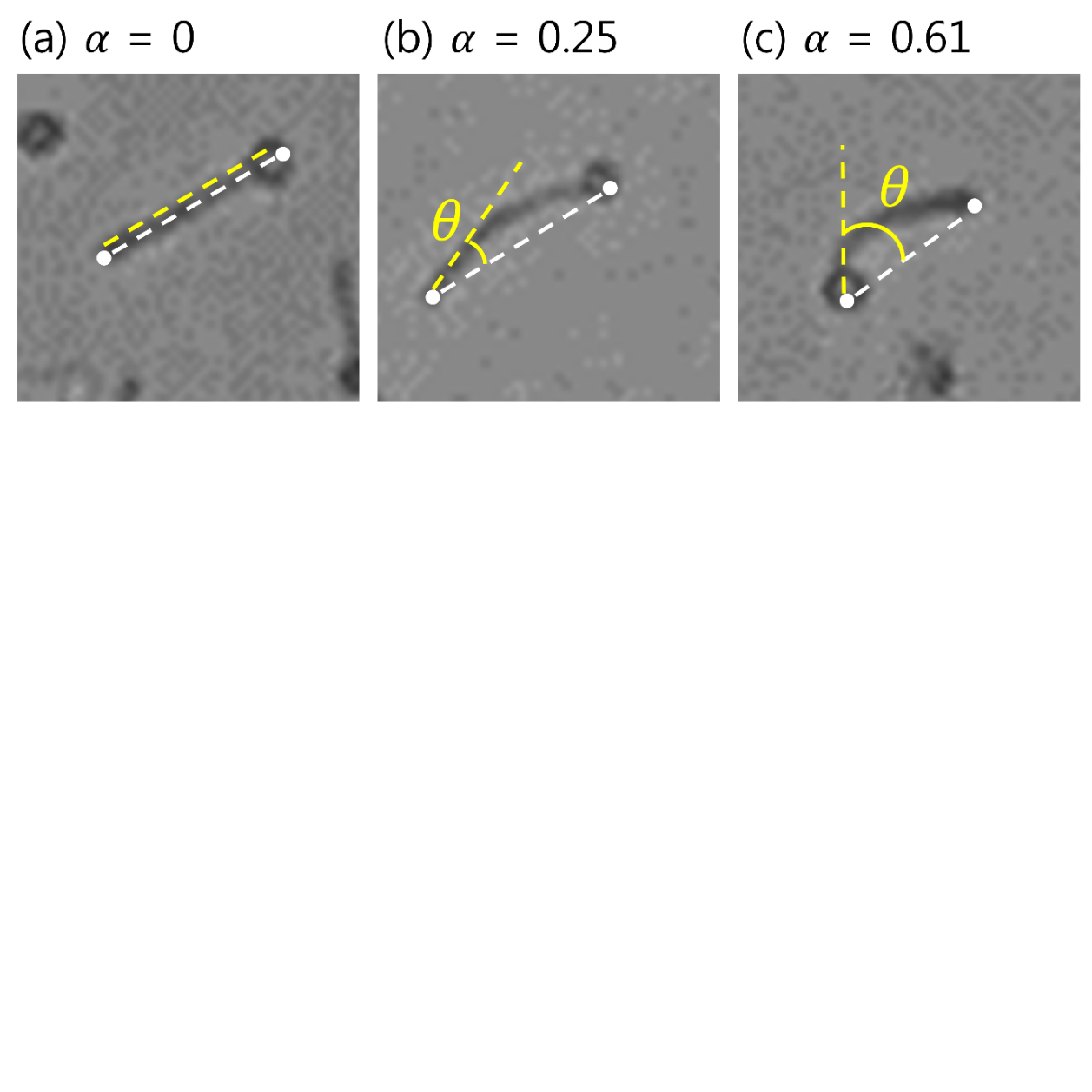


**Figure S1.** Asymmetrical coefficient (𝛼) of 3-h-incubated candida cells (**a**) 𝛼 = 0, (**b**) 𝛼 = 0.25, and (**c**) 𝛼 = 0.61. The white dotted line shows a straight line connecting two points located at each end of the candida cells, and the yellow dotted line depicts a tangent line at the end of the germ tube.

As *C. albicans* is incubated in human serum or rabbit serum at 37 °C for 2–3 h, short germ tubes are induced from the mother yeast cell. Germ tubes grown from the cell have random curvature, as shown in Figure 2 and Figure S1. The asymmetrical shape of *C. albicans* affects the initialization of cells at the first bifurcation before the separation process and even affects the separation efficiency. Therefore, to characterize the asymmetry of *C. albicans*, an asymmetrical coefficient (𝛼) was adopted. We measured the angle (𝜃) between a straight line connecting two points located at each end of candida cells and a tangent line at the end of the germ tube (Figure S1). The asymmetrical coefficient (𝛼) of *C. albicans* was defined as the angle (𝜃) divided by 90°:

| $\alpha=\frac{\theta}{90^{\circ}}$. | (1) |
| --- | --- |

The three-hour-incubated candida cells has $0\leq\alpha\leq0.7$, and approximately 40% of the 3-h-incubated candida cells had a asymmetrical coefficient higher than 0.3, which affected the initialization at the first bifurcation and the random distribution across the channel in the expansion region.

1. Flow Characteristics of Three-Hour-Incubated *Candida* Cells


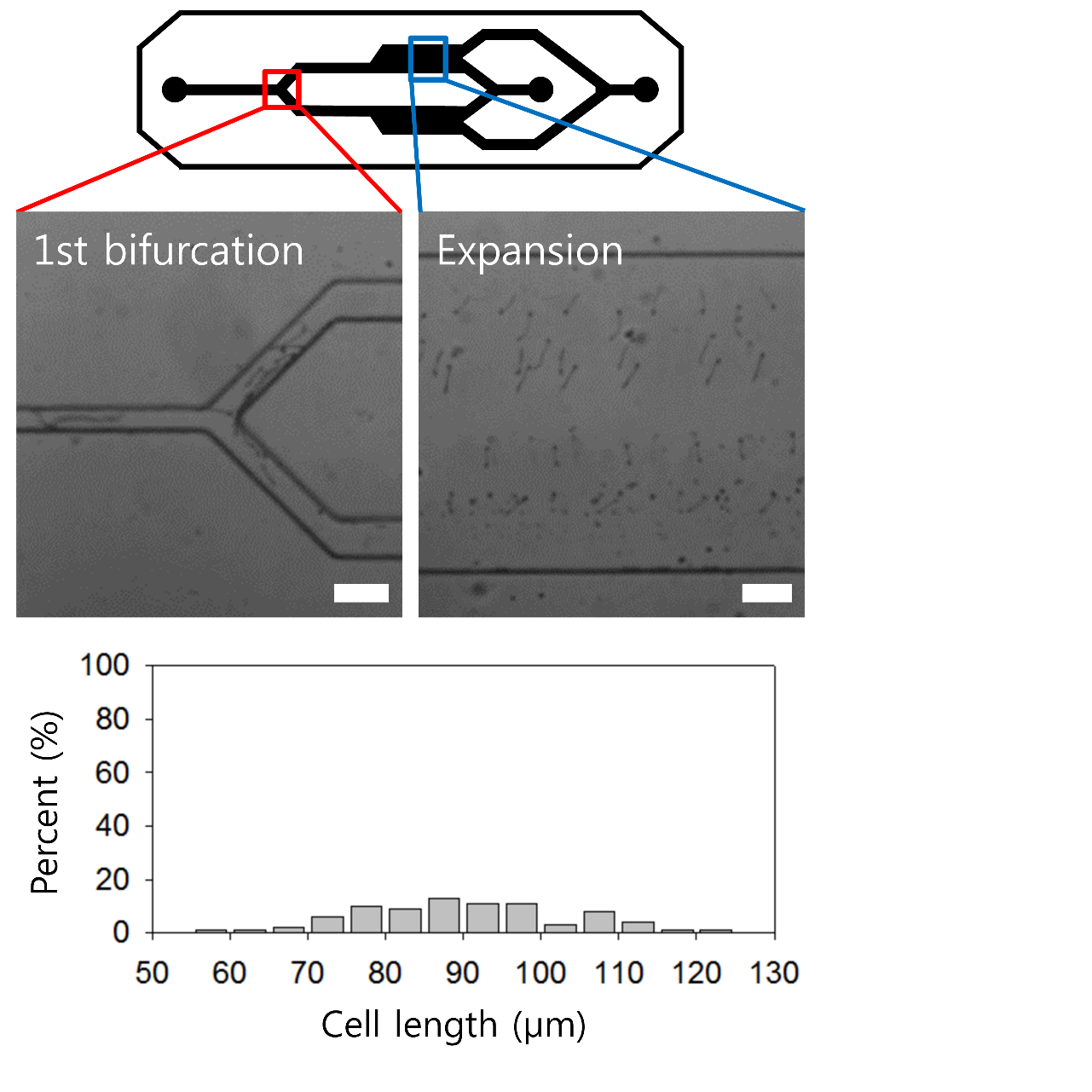


**Figure S2.** Flow characteristics of candida cells incubated for 3 h at 37 °C at the first bifurcation and the second bifurcation at *Q* = 50 μL/min. The scale bars are 100 μm. Size distribution of candida cells incubated for 3 h at 37 °C.

The 3-h-incubated *C. albicans* had a diameter of 90.03 ± 13.26μm, which became longer than the width of the first-stage microchannel (40 μm). As shown in Figure S2, most 3-h-incubated candida cells were not focused at the centerline at a flow rate of 50 μL/min, while spherical candida cells were focused at the centerline of the microchannel, which was speculated to have been due to the asymmetric orientation of 3-h-incubated candida cells. The cells were randomly distributed at the first bifurcation and widely spread over the channel width in the expansion region, even beyond the centerline of the microchannel. Therefore, the use of 3-h-incubated candida cells was found to be not suitable for the working principle that initialized the positions of all particles/cells at the first bifurcation and separated them according to shape-dependent viscoelastic lateral migration in the second-stage microchannel.
